# 

**Published:** 1901-12

**Authors:** 


### The Library of the Bristol Medico-Chirurgical Society.

*The following donations have been received since the publication of the List in September:*

*November 30th, 1901.*

| | |
|---|---|
| J. Michell Clarke, M.D. (1) ... ... ... ... ... | 3 volumes. |
| L. M. Griffiths (2) ... ... ... ... ... ... ... | 14 " |
| The Representatives of the late J. E. Prichard, M.B. ... ... ... ... ... ... ... ... ... | 1 volume. |
| S. T. Pruen, M.D. (3) ... ... ... ... ... ... | 2 volumes. |
| Robert Roxburgh, M.B. ... | Complete set of *Virchow's Archiv.* |
| The Council of University College, Bristol (4) ... | 1 volume. |
| The Editor of the "Virginia Medical Semi-Monthly" (5) ... ... ... ... ... ... ... | 1 " |

### FORTY-SECOND LIST OF BOOKS.

The titles of books mentioned in previous lists are not repeated.

The figures in brackets refer to the figures after the names of the donors, and show by whom the volumes were presented. The books to which no such figures are attached have either been bought from the Library Fund or received through the *Journal.*

| | | |
|---|---|---|
| **Agote et A. J. Medina, L.** | *La Peste bubonique dans la République Argentine et au Paraquay—Épidémies de* 1899—1900 ... ... | 1901 |
| **Allen, B.** ... ... | *The Natural History of the Chalybeat and Purging Waters of England* ... ... ... ... ... ... ... ... | 1699 |
| **Ball, J. B.** ... ... | *Diseases of the Nose and Pharynx* ... ... ... 4th Ed. | 1901 |
| **Bastian, H. C.** ... | *Studies in Heterogenesis.* Part I. ... ... ... ... ... | 1901 |
| **Black, W. G.** ... | *Folk-Medicine* ... ... ... ... ... ... ... ... ... ... | 1883 |
| **Carpenter, G.** ... | *Golden Rules for Diseases of Children* ... ... ... ... | [1901] |
| **Carter, A. H.** ... | *Elements of Practical Medicine* ... ... ... 8th Ed. | 1901 |
| **Celsus** ... ... ... | *De Re Medica Libri Octo ex recensione L. Targæ.* (Ed. by G. F. Collier) ... ... ... (2) 3rd Ed. | 1901 |
| " ... ... ... | *A Translation of the Eight Books on Medicine.* (Ed. by G. F. Collier) ... ... ... ... ...(2) 3rd Ed. | 1838 |
| **Cervello, V.** ... | *On the Cure of Pulmonary Tuberculosis* ... ... ... ... | 1899 |
| *Cheltenham Ladies' College, Guide to the* ... ... ... ... ... ... (3) | | [N.D.] |
| *Contradiction : or, English Medical Men and Manners of the Nineteenth Century* ... ... ... ... ... ... ... (2) | | 1877 |
| **Crocq, J.** ... ... | *Étude sur l'Adénie ou Pseudo-Leucémie* ... ... ... (1) | 1891 |
| *Doctors, My. By a Patient* ... ... ... ... ... ... ... ... (2) | | 1891 |
| **Dupouy, E.** ... | *Medicine and Morals of Ancient Rome according to the Latin Poets.* (Tr. by T. C. Minor) ... ... ... | 1901 |
| **Faraday, [M.]** ... | *On the Non-Metallic Elements.* (Ed. by J. Scoffern) (2) | 1853 |
| **Fernie, W. T.** ... | *Kitchen Physic* ... ... ... ... ... ... ... ... ... ... | 1901 |

**Garrett, J. H.** ... *Cheltenham and the Cheltenham Country* ... ... (3) 1901
**Giles, A. E.** ... *Menstruation and its Disorders* ... ... ... ... ... ... 1901
**Harrison, R.** ... *Renal Tension and its Treatment by Surgical Means* [1901]
**Hughes, A. W.** ... *Practical Anatomy.* Part I. (Ed. and completed by A. Keith) ... ... ... ... ... ... ... ... ... ... 1901
**Jeaffreson, J. C.** *A Book about Doctors* ... ... ... ... ... ... ... ... ... [N.D.]
**Jellett, H.** ... ... *A Short Practice of Midwifery* ... ... ... 3rd Ed. 1901
**Jennings, O.** ... *The Morphia Habit* ... ... ... ... ... ... ... 2nd Ed. 1901
**Jones, H. B.** ... *Croonian Lectures on Matter and Force* ... ... ... (2) 1868
**Jones, H. M.** ... *Practical Points in Gynæcology* ... ... ... 2nd Ed. 1901
**Jones, L. V.** ... *Gonorrhœal Arthritis* ... ... ... ... ... ... ... ... ... 1901
**Julien, L.** ... ... *Libertinism and Marriage* (Tr. by R. B. Douglas) ... 1901
**Madden, F.C.** ... *The Practical Nursing of Infants and Children* .. ... 1901
**Markham, W. O.** *Diseases of the Heart* ... ... ... ... ... ... ... (2) 1856
**Medina, L. Agote et A. J.** *La Peste bubonique dans la République Argentine et au Paraquay—Épidémies de* 1899—1900 ... ... 1901
**Méric, H. de** ... *Syphilis and other Venereal Diseases* ... ... ... ... 1901
**Mortimer, J. D. E.** *Tendencies to Consumption* ... ... ... ... ... ... [N.D.]
**Moynihan, A. W. M. Robson and B. G. A.** *Diseases of the Stomach and their Surgical Treatment* ... ... ... ... ... ... 1901
*Paget, Memoirs and Letters of Sir James.* (Ed. by S. Paget) 2nd Ed. 1901
**Palmer, Margaret D.** *Lessons on Massage* ... ... ... ... ... ... ... ... 1901
**Peters, J. A.** ... *Massage* ... ... ... ... ... ... ... ... ... ... ... ... ... 1901
*Pharmacopœia of the Hospital for Diseases of the Throat, Nose, and Ear, The* ... ... ... ... ... ... ... ... ... 6th Ed. 1901
**Robson and B. G. A. Moynihan, A. W. M.** *Diseases of the Stomach and their Surgical Treatment* ... ... ... ... ... ... ... 1901
*Royal College of Surgeons of England, Synopsis of the Contents of the Museum of the* ... ... ... ... ... ... ... ... (2) 1867
**Ruata, C.** ... ... *Pulmonary Tuberculosis* ... ... ... ... ... ... ... ... ... 1901
**Russell, J. R.** ... *The History and Heroes of the Art of Medicine* ... ... 1861
**Schaeffer, R.** ... *Beiträge zur Händedesinfectionsfrage* ... ... ... 1902 [*sic.*]
**Stacpoole, Florence.** *Advice to Women* ... ... ... ... ... ... 3rd Ed. 1901
**Strümpell, A.** ... *A Text-Book of Medicine.* (3rd American Edition, Tr. from the 13th German. Ed. by H. F. Vickery and P. C. Knapp) ... ... ... ... ... ... 1901
**Sydenham, T.** ... *Works.* (Tr. by J. Swan) ... ... ... (2) 2nd Ed. 1749
**Taylor, A.** ... ... *The Sanitary Inspector's Handbook* ... ... ... 3rd Ed. 1901
**Treves, Sir F.** ... *Surgical Applied Anatomy* ... ... New [4th] Ed. 1901
*Turkish Bath Question, The* ... ... ... ... ... ... ... ... (2) 6th Ed. [N.D.]
**Turner, A. L.** ... *The Accessory Sinuses of the Nose* ... ... ... ... ... 1901
**White, W. H.** [Ed.] *Text-Book of Pharmacology and Therapeutics* . ... 1901
**Winter, G.** ... *Animal Magnetism* ... ... ... ... ... ... ... ... ... (2) [1801]

### TRANSACTIONS, REPORTS, JOURNALS, &c.

American Association of Obstetricians and Gynecologists, Transactions of the ... ... ... ... ... ... ... ... ... ... ... Vol. XIII. 1901
American Gynæcological and Obstetrical Journal, The ... Vol. XVIII. 1901
American Journal of Obstetrics, The ... ... ... ... ... ... Vol. XLIII. 1901
American Medicine ... ... ... ... ... ... ... ... ... ... ... ... Vol. I. 1901
Archiv für pathologische Anatomie und Physiologie und für klinische Medicin ... ... ... ... ... ... ... ... Bd. I.—CLXVI., 1847—1901

Archivio di Ortopedia ... ... ... ... ... ... ... ... ... ... ... ... 1900
Army Regulations, Vol. VI.—Regulations for the Medical Department (2) 1885
Boston Medical and Surgical Journal, The ... ... ... Vol. CXLIV. 1901
Buffalo Medical Journal ... ... ... ... ... ... ... ... Vol. XL. 1901
Catalogue of Books, The English ... ... ... Vol. IV. (1898—1900) 1901
China Medical Missionary Journal, The ... Vols. XIII., XIV. 1899—1900
Cincinnati Lancet-Clinic, The ... ... ... ... ... Vol. LXXXV. 1901
Clinical Society of London, Transactions of the ... Vol. XXXIV. 1901
Congrès (XIII[e]) international de Médecine, Paris, 1900.—Comptes Rendus ... ... ... ... ... ... [Vols. II., VIII., XV., XVI. 1901]
Edinburgh Obstetrical Society, The Transactions of the Vol. XXVI. 1901
Grant College Medical Society, Bombay, Transactions of the, 1900 ... 1901
Glasgow Medical Journal, The ... ... ... ... ... ... Vol. LV. 1901
Hospitals—
Boston City Hospital, Medical and Surgical Reports of the (1) 1898, 1900
Guy's Hospital Reports ... ... ... ... ... ... Vol. LV. 1901
Middlesex Hospital Reports, 1898 ... ... ... ... ... ... ... 1900
Westminster Hospital Reports, The ... ... ... ... Vol. XII. 1901
Journal of the Boston Society of Medical Sciences ... ... Vol. V. 1900-01
Journal of the Sanitary Institute, The ... ... ... ... ... Vol. XXI. 1900
Library Association Year Book, The ... ... ... ... ... ... ... ... 1901
Library World, The ... ... ... ... ... ... ... ... ... ... Vol. III. 1901
Louisville Monthly Journal, The ... ... ... ... ... ... Vol. VII. 1900-01
Medical News, The ... ... ... ... ... ... ... ... ... Vol. LXXVIII. 1901
Medical Press and Circular, The ... ... ... ... ... [Vol. CXXII.] 1901
New York Medical Journal, The ... ... .. ... ... Vol. LXXIII. 1901
New York, Transactions of the Medical Society of the State of ... ... 1901
Norsk magazin for lægevidenskaben, 3[die] hovedregister for. Aargangen 1871—1900 ... ... ... ... ... ... ... ... ... ... ... ... ... 1901
Polyclinic, The ... ... ... ... ... ... ... ... ... ... ... ... Vol. IV. 1901
Practitioner, The ... ... ... ... ... ... ... ... ... ... Vol. LXVI. 1901
Scottish Medical and Surgical Journal, The ... ... ... Vol. VIII. 1901
Sei-I-Kwai Medical Journal, The ... ... ... ... ... ... Vol. XIX. 1900
Smithsonian Report, 1899 ... ... ... ... ... ... ... ... ... ... ... 1901
University College, Bristol.—Calendar, 1901—1902 ... ... ... ... (4) 1901
Virginia Medical Semi-Monthly ... ... ... ... ... ... ... (5) Vol. V. 1901
Virchow's Archiv. *See* Archiv für pathologische Anatomie [etc.]
Year-Book of the Scientific and Learned Societies ... ... ... ... ... 1901

### MEETINGS OF SOCIETIES.

### Bristol Medico-Chirurgical Society.

ANNUAL MEETING, *October 9th, 1901.*

After a vote of thanks to the retiring President (Dr. D. S. Davies) had been unanimously passed, Dr. BARCLAY J. BARON took the chair and gave the introductory address (see p. 289), for which a cordial vote of thanks was afterwards given.

The HONORARY SECRETARY (Mr. J. Paul Bush) read his Annual Report. He had to record that the Society had lost three of its

### Local Medical Notes.

### BRISTOL.

University College, Bristol.—George Parker, M.A., M.D. Cantab., has been appointed Physician, and Newman Neild, M.B., B.Ch. Vict., Assistant Physician, to the Bristol General Hospital. J. Paul Bush, C.M.G., has been appointed President of the United Kingdom Police Surgeons' Association. At the Royal Infirmary, Mr. E. C. Mackay, M.R.C.S., L.R.C.P., has been appointed Anæsthetist and Junior House Surgeon, and Mr. H. E. G. Boyle, M.R.C.S., L.R.C.P., Casualty Officer. At the Bristol General Hospital, Mr. E. R. Clark, M.B., B.Ch. Cantab., has been appointed Assistant House Surgeon, and Mr. J. E. Sparks, M.R.C.S., L.R.C.P., Casualty House Surgeon. In the Intermediate M.B. Examination of the London University, Messrs. J. D. Calcott, A. W. C. Richardson, and J. B. V. Watts were successful candidates. Mr. J. Phillips was successful in the Final F.R.C.S. Edin. Examination, and Messrs. J. C. Clayton, A. Coleridge, H. James, and J. E. Sparkes were successful in the Final M.R.C.S. Eng. and L.R.C.P. Lond. Examinations. At the Hospital for Consumption, Hampstead, London, N.W., Messrs. H. S. Jenkins, M.B. Lond., and S. R. Williams, M.R.C.S., L.R.C.P., have been appointed respectively Senior and Junior Resident Medical Officers.

Bristol Royal Infirmary.—Some of the principal Alterations and Improvements made at the Bristol Royal Infirmary during the past Ten Years.—For these notes we are indebted to Mr. Edward A. Leonard, the Secretary. In 1892 *Isolation Wards* for infectious diseases were built in the garden; in 1893 an *Obstetric Ward* was built at the top of the house where the nurses' dormitories had been vacated by the removal of their occupants to the Nurses' Home. In 1894 and 1895 the *Out-patient Department* was greatly extended and improved; and in 1896 a re-arrangement of some of the rooms made it possible to provide a *New Casualty Room*, larger and more conveniently situated than its predecessor. In 1897 there was a general *Renovation of the Wards*, and *New Sanitary Appliances* were provided in all parts of the building. In the same year the reconstruction of the *Operation Theatres* was commenced; and the completion of this work as a memorial to the late Mr. Greig Smith was a very protracted and expensive undertaking. In 1899 an important addition to the *Nurses' Home* was made by the erection of a large new wing, and the removal of the nurses from the old building to the enlarged new home made it possible to carry out other improvements, such as the provision of a comfortable ward for nurses who might be ill, also a cloak-room, lavatory, and sitting-room for their use. In this year also the *Steam Laundry* was much enlarged by the construction of a new storey; and it was improved by the provision of additional machinery, making it possible for the staff to wash some seven thousand articles per week. In 1898 an *Honorary Skiagraphist* was appointed; and three rooms have been fitted up for his department, one of these being a photographic dark room provided with the requisite apparatus. Great improvements have recently been effected in the Museum, the kitchens, the mortuary; and a general

*re-construction of the house drainage system* is now being carried on, which will provide effectual means for inspection, cleansing and ventilation of the drains.

### NEWPORT.

Description of the New Hospital.—The architecture of the building is Renaissance, and is faced with deep red bricks. The window dressings and horizontal bands are of Bath stone.

The Hospital has been designed on the pavilion principle, with the administrative block in the centre, and the accommodation provides for 84 patients.

The floors of the large wards are narrow pitch-pine boards, nailed to wood fillets set in fire-proof floor. The wards are heated by means of ventilating hot-water radiators on the low pressure system; and, in addition, each ward has a double central fireplace with descending flues. The sanitary fittings are of the latest approved patterns which scientific sanitary engineers have invented.

The operating theatre is placed close to the patients' entrance in the rear of the main corridor, with direct communication from same, and is provided with abundance of light from the sides, in addition to a large ventilating lantern light. The internal fittings of the operating theatre are all of special patterns, such as are now used in the best of the modern hospitals. Two white enamelled porcelain sponge sinks with marble trays, electro-plated wringer and service taps (hot and cold water) are provided on one side. The radiators are so fixed that the cold air supply is warmed as it enters the theatre.

Adjoining the theatre on one side is the anæsthetic room, in which are fixed the sterilizers on polished plate-glass shelves. On the other side is the hydraulic lift, known as the "Reliance," capable of taking a patient on a bed and an attendant to either of the two floors above. To work this lift a special water main has been laid on by the Corporation, connecting the high pressure mains on Stow Hill with the Hospital.

The administrative block, which forms the central feature of the Hospital, is devoted to the use of the executive and officials. It is entered from the terrace by a wide flight of steps. Passing through the outer vestibule and on through a glazed screen, the handsome entrance hall, 21 feet by 15 feet, is reached.

The whole building is constructed on the principle of a fire-proof building, and the various floors are all connected by means of a special fire alarm system to a central spot on the ground floor. In the event of fire breaking out in any portion of the building, on going to the nearest alarm post, of which there are two on every floor, a bell is rung on every floor, showing where the fire is, and the bells continue ringing until the attendant at the central spot has noticed the same.

The out-patient department has been built as a detached and distinct building fronting Cardiff Road, close to the main entrance to the Hospital grounds. The out-patients on entering, after passing the registration offices, enter a large waiting hall, on either side of which are arranged the medical, surgical, and ophthalmic consulting rooms. The internal arrangement of this block permits the patients, after being examined in one of the consulting rooms, to pass directly to the dispensary, obtain their medicine, and leave the building without returning through the waiting hall.

The in-patients' entrance is placed at the eastern end of the main corridor, and is approached from Cardiff Road by a carriage drive 16 feet wide. Adjoining the entrance on the right is the in-patients'

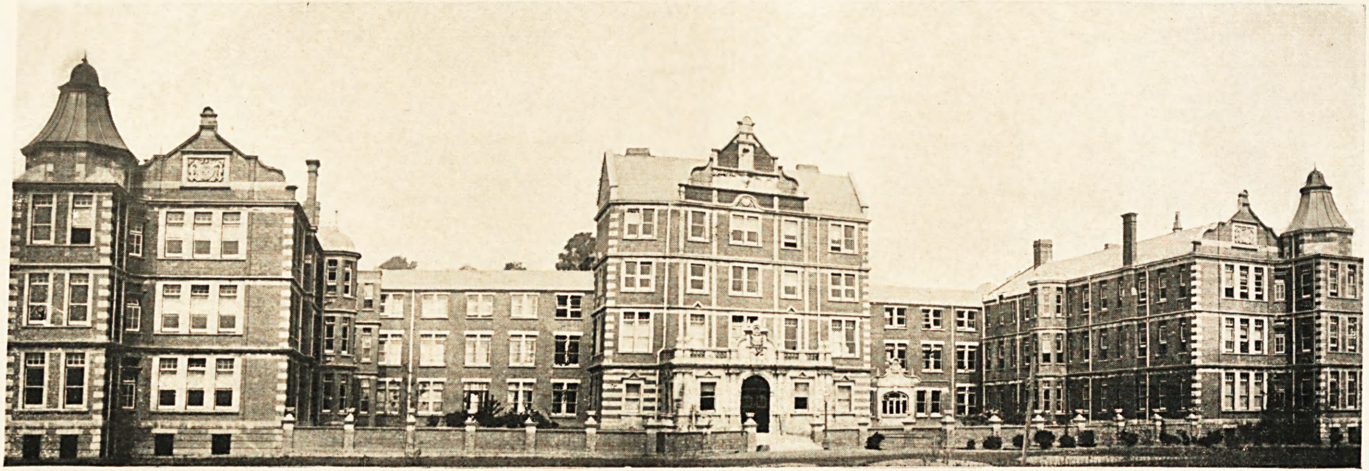

NEWPORT AND MONMOUTHSHIRE NEW HOSPITAL.

receiving room, where the patient is examined prior to being sent on to one of the wards.

The laundry and boiler house is a separate and detached building, situate at the rear of the main buildings.

In the basement under the centre of the administrative block are fixed the electrical transformers, the calorifiers for heating the whole of the buildings and those for the hot-water supply; the steam from main boilers being utilized to heat these calorifiers. Spacious storage rooms are also arranged in the basement.

The whole of the Hospital buildings and roadways are to be lit by electricity, supplied from the mains of the Newport Corporation.

The electric light installation consists of about 600 incandescent lamps and an arc lamp at the main entrance. The whole system of lighting is controlled from a main switchboard fixed in the centre of the ground-floor corridor, from whence sub-mains are led to fuse-distribution boards supplying the lamps of the various portions of the installation, each portion being thoroughly under control. The wiring is so arranged that the system can be easily tested, every eight lamps being controlled by a double-pole fuse on the sub-distribution boards.

In the corridors the lamps are arranged alternately on two circuits, with controlling switches to enable the amount of light to be varied.

In the six wards the lighting is arranged by means of two 3-light pendants at the ends and one 2-light pendant in the centre, each lamp being frosted so as to give a soft light. In addition a dimming coil apparatus is provided, by means of which it is possible to reduce the light of one lamp in each of the 3-light fittings from 16 candle-power to about 2 or 3 candle-power, to provide a dim light during the night when all the other lamps are extinguished. Between the beds, on the wall, plug connections have been arranged to enable portable lamps to be used for special examinations, etc., at night. These fittings may, if necessary, be hung on the wall over the bed, and a similar fitting also serves the purpose of lighting the night nurse's table.

The operating theatre will be provided with one of the latest pattern of adjustable fittings to turn the light at any angle on to the operating table. It is contemplated to fit up an X-ray apparatus and a suitable supply of electro-cautery devices. The electrical installation has necessitated the use of 2¾ miles of conduit and 8½ miles of insulated cables and wires.

A complete gas installation has been laid on throughout the whole of the buildings for use in case of a failure in the electric light, or breakdown of any portion of the electrical machinery.

---

### Obituary.

### Dr. KENDALL.

From the *Western Morning News* of October 25th, 1901, we obtain the following particulars relating to the death of Dr. Kendall at Helston, at the age of 36:—"Very few but the most intimate friends of the family were aware that he had been indisposed for a day or two. He was away for several hours on Monday night attending a serious

### LIST OF SUBSCRIBERS

TO

### The Bristol Medico-Chirurgical Journal.

*DECEMBER*, 1901.

---

### England.

### CAMBRIDGESHIRE.

CAMBRIDGE.
G. S. Woodhead

WALPOLE ST. ANDREW.
R. Smith

### CHESHIRE.

ALDERLEY EDGE.
J. McElfatrick

NEW BRIGHTON.
A. W. Riddell

### CORNWALL.

CAMBORNE.
T. C. Grey

PENZANCE.

W. S. Bennett

J. A. Fox

J. Symons

LISKEARD.
S. E. Rigg

ST. AUSTELL.
W. Mason

### DEVONSHIRE.

BABBACOMBE.
T. Finch

BAMPTON.
A. R. Down

BARNSTAPLE.
C. H. Gamble
J. Harper
M. Jackson

BRIXHAM.
G. C. Searle

BROAD CLYST.
J. Somer

BUDLEIGH SALTERTON.
T. G. C. Evans

CHAGFORD.
A. D. Hunt

CULLOMPTON.
G. G. Gidley

DARTMOUTH.
R. B. Searle

EXETER.
J. M. Ackland
H. Davy
R. V. Solly
E. B. Steele-Perkins
R. Thomas
L. H. Tosswill

NEWTON ABBOT.
J. Culross

### DEVONSHIRE *(Continued).*

PAIGNTON.

J. Alexander

PLYMOUTH.

G. F. Aldous
R. H. Lucy
Medical Society
E. B. Thomson
W. L. Woollcombe

PLYMPTON.

C. Aldridge
W. D Stamp

SOUTH MOLTON.

H. J. Smyth

TEIGNMCUTH.

T. E. Frazer-Toovey

TORQUAY.

W. Odell
R. Pollard
W. Powell
C. H. Wade

WINKLEIGH.

J. H. Norman

### DORSETSHIRE.

DORCHESTER.

P. W. MacDonald

WEYMOUTH.

J. M. Lawrie

### ESSEX.

NEWPORT.

W. A. Smith

WALTHAMSTOW.

F. P. Elliott

### GLOUCESTERSHIRE.

ALMONDSBURY.

J. C. McWatters

BRISTOL.

W. R. Ackland
J. Ambrose
W. M. Barclay
T. G. L'E Baretti
G. H. Barker
B. J. Baron
A. E. Blacker
A. F. Blagg
C. W. J. Brasher
H. F. Briggs
F. St. J. Bullen
E. G. Bunbury
E. F. H. Burroughs
J. P. Bush
A. Carr
T. M. Carter
T. Carwardine
R. H. Chilton
J. M. Clarke
T. V. Coker
H. Cook
W. Cotton
R. J. Coulter
F. R. Cross
J. Dacre
D. S. Davies
N. C. Dobson
E. L. W. Dunbar
E. Eberle
F. H. Edgeworth
W. Elder
C. Elliott
E. Fawcett
R. G. Fendick
J. L. Firth
T. Fisher
P. Flood
E. V. Foss
E. L. Fox
J. Freeman
E. B. Garland
T. T. Genge
W. C. Gent
D. S Gerrish
A. N. G. Gibbs
J. S. Griffiths
L. M. Griffiths
C. D. G. Hailes
A. H. Haines
H. E. Harris
A. J. Harrison
W. H. Harsant
C. A. Hayman
Miss Heath
J. C. Heaven
C. K. C. Herapath
H. E. Hetling
H. Hill
T. Hill
General Hospital
G. A. Imlay
H. W. Kendall
F. P. Lansdown
R. G. P. Lansdown
E. L. Lees
R. C. Leonard
W. Ligertwood
J. J. S. Lucas
P. W. McCrea
A. R. McKee
G. Metcalfe
H. F. Mole
A. T. Morgan
C. A. Morton
G. T. Myles
N. Neild
W. N. Nevill
C. Newman
W. H. C. Newnham
T. D. Nicholson
C. O'Brien
A. Ogilvy
G. S. Page
G. Parker
J. H. Parry
A. W. Peake
W. A. Perry
C. F. Pickering
W. E. Pountney
J. J. Powell
A. W. Prichard
A. B. Prowse

### GLOUCESTERSHIRE *(Continued)*.

### BRISTOL *(continued)*.

D. C. Rayner
B. M. H. Rogers
F. H. Rose
W. B. Roué
C. K. Rudge
H. T. Rudge
J. E. Shaw
E. J. Sheppard
E. M. Skerritt
G. M. Smith
R. S. Smith
E. H. E. Stack
J. D. Staple
C. Steele
W. H. Steele
J. B. Stephenson
W. H. Stevens
S. V. Stock
F. W. Stoddart
J. Swain
J. G. Swayne
W. C. Swayne
J. O. Symes
J. Taylor
W. J. Tivy
H. Waldo
C. H. Walker
Canon Wallace
J. Wallace
J. H. Wathen
J. B. Webb
E. C. Williams
P. W. Williams
W. R. Williams
W. K. Wills
H. W. Windsor-Aubrey
C. Wintle

### CHELTENHAM.

W. J. K. Millard
G. A. Peake
E. Trevithick

### CHIPPING SODBURY.

F. F. Fox
A. Grace

### CIRENCESTER.

W. R. Cossham
O. H. Fowler
C. Mackinnon

### DOWNEND.

H. Skelton

### FISHPONDS.

H. A. Benham
C Bernard
W. Brown
A. L. Flemming

### FYLTON.

F. P. Mackie

### GLOUCESTER.

R. W. Batten
W. R. Hadwen
W. Hodges

### HAMBROOK.

E. Crossman
F. W. Crossman
W. F. B. Eadon

### KINGSWOOD.

E. W. H. Groves
L. W. Powell

### LYDNEY.

W. P. Kennedy

### REDFIELD.

J. W. Rodgers

### ST. GEORGE.

J. Young

### STOKE BISHOP.

H. F. Parsons.

### STONEHOUSE.

G. T. B. Watters

### STOW-ON-THE-WOLD.

E. Dening

### STROUD.

A. S. Cooke

### TETBURY.

W. Wickham

### THORNBURY.

E. M. Grace
L. H. Williams

### WESTBURY-ON-TRYM.

A. Harvey
H. L. Ormerod

### WINTERBOURNE.

R. Eager
W. R. Eager

### WOTTON-UNDER-EDGE

J. G. Boyce
D. H. Forty

### HAMPSHIRE.

### BOURNEMOUTH.

C. H. Ackland
J. S. Dickie
J. G. Harsant

### CARISBROOKE.

J. Groves

### FLEET.

H. Wilcox

### LYMINGTON.

J. Rendall

### SOUTHAMPTON.

W. R. Y. Ives

### SOUTHSEA.

J. W. Cousins

### LANCASHIRE.

### LIVERPOOL.

G. A. Hawkins-Ambler

### MANCHESTER.

Bayer Co., Ltd.
Medical Society

### MIDDLESEX.

### LONDON.

J. Berry
T. C. Fox
M. Handfield-Jones
W. H. A. Jacobson
H. M. Jones
Lord Lister
J. Macready
A. L. Marshall
F. T. Roberts
W. J. Seward
J. A. Shaw-Mackenzie

### TOTTENHAM.

W. H. Plaister

### TWICKENHAM.

A. E. Neale

### MONMOUTHSHIRE.

### NEWPORT.

W. Basset
C. B. Gratte
W. J. Greer
O. E. B. Marsh
A. G. Thomas
H. E. Williams

### PONTYPOOL.

J. R. Essex
S. B. Mason

### RHYMNEY.

T. H. Redwood

### TREDEGAR.

G. A. Brown

### NOTTINGHAMSHIRE.

### NOTTINGHAM.

C. B. Taylor
W. M. Willis

### OXFORDSHIRE.

### OXFORD.

H. P. Symonds

### SOMERSETSHIRE

### BATH.

W. D. Akers

E. J. Cave

F. S. Cowan

S. Craddock

W. M. Ellis

T. B. Goss

F. Lace

T. P. Lowe

G. J. K. Martin

W. S. Melsome

R. J. H. Scott

L. H. Walsh

L. A. Weatherly

J. Wigmore

A. S. Wohlmann

### BRIDGWATER.

F. J. C. Parsons
R. H. F. Routh

### CHEDDAR.

E. H. Openshaw
R. W. Statham

### CREWKERNE.

W. W. Webber

### BRISLINGTON.

B. B. Fox
W. B. Morton
C. M. Phillips

### CLEVEDON.

T. Davis

W. J. Hill

### CURRY RIVEL.

R. H. Vereker

### BURNHAM.

F. C. Berry

### COMPTON MARTIN.

H. C. Linden

### FRESHFORD.

C. E. S. Flemming

### SOMERSETSHIRE *(Continued)*.

FROME.

A. W. Dalby
A. G. Hayman
J. M. Rattray
C. R. Wood

GLASTONBURY.

J. C. Smyth

HAM GREEN.

J. Fletcher

ILMINSTER.

C. Munden

KEYNSHAM.

G. G. D. Willett

LANGPORT.

J. Morgan

MILVERTON.

C. Randolph

NAILSEA.

J. W. White

PILL.

T. W. S. Morgan

PORTISHEAD.

G. S. J. Boyd
W. Monckton
C. A. Wigan

TAUNTON.

H. T. S. Aveline
Taunton and Somerset Hospital
H. T. Rutherford

TIMSBURY.

D. L. Hamilton

WEDMORE.

J. Ford
R. P. Tyley

WELLINGTON.

J. Meredith

WELLS.

W. Fairbanks
W. A. L. Smith
C. J. Whitby

WEST COKER.

G. C. Wilkin

WESTON-SUPER-MARE.

H. T. M. Alford
H. S. Ballance
C. P. Crouch
E. F. Martin
G. F. Rossiter
R. Roxburgh
G. H. Temple
J. Wallace

WORLE.

F. St. J. Kemm

WRINGTON.

H. C. Bristowe
H. W. Collins

YEOVIL.

W. R. M. Semple

### WARWICKSHIRE.

LEAMINGTON.

A. W. W. Hoffman

### WILTSHIRE.

BRADFORD-ON-AVON.

W. J. A. Adye
J. Beddoe

CALNE.

W. S. Batten

CORSHAM.

J. E. Crisp

FOVANT.

C. Clay

MALMESBURY.

R. Kinneir

SWINDON.

J. C. Maclean

WARMINSTER.

S. V. Theed
R. L. Willcox

### WORCESTERSHIRE.

WORCESTER.

R. W. Brimacombe

### Ireland.

### ANTRIM.

BELFAST.

J. W. Browne

A. Jamison

J. A. Lindsay

### Wales.

### BRECONSHIRE.

BRECON.

W. Howells

### CARMARTHENSHIRE.

FERRYSIDE.

P. Williams

LLANELLY.

S. J. Roderick

### GLAMORGANSHIRE.

ABERDARE.

E. Jones

CARDIFF.

W. J. Corrigan
P. R. Griffiths
T. G. Horder
E. J. Maclean
D. R. Paterson
A. Sheen
Medical Society
J. L. Thomas
H. R. Vachell

GORSEINON.

T. Mitchell

MERTHYR TYDVIL.

W. W. Jones
J. L. W. Ward

PENARTH.

C. E. Matthews

SWANSEA.

E. Le C. Lancaster

TREHARRIS.

W. W. Leigh

### PEMBROKESHIRE.

HAVERFORDWEST.

E. P. Phillips

### France.

PARIS.

H. Le Soudier

### Switzerland.

DAVOS-PLATZ.

W. R. Huggard

### Cape Colony.

FORT BEAUFORT.

J. Shaw

KING WILLIAM'S TOWN.

H. M. Chute

### United States.

CHICAGO.

Columbus Medical Library

COLORADO.

S. E. Solly

PHILADELPHIA.

Philadelphia Medical Journal.

### Italy.

GENOA.

S. Biamonti

### Egypt.

CAIRO.

M. A. Ruffer

### New Zealand.

LOWER HUTT.

J. R. Purdy

### India.

BHAGALPORE.

N. Muzumidar

CALCUTTA.

Major Peck

### INDEX.

(R.) = Review.

---

Abdominal operations, Retrospect of a third series of fifty consecutive intra-—Dr. J. Swain, 18, 121.
Abdominal suppuration, Clinical lectures on intra-—A. H. Tubby (R.), 350.
Abdominal viscera, Some cases of displacement of—Dr. T. Fisher, 210.
Abrams, A.—Transactions of the antiseptic club (R.), 272.
Abscess in the left lateral lobe of the cerebellum, Case of—Dr. J. M. Clarke and C. A. Morton, 112.
Actinomycosis, 55.
Albuminuria, Cyclic — Dr. G. A. Sutherland (R.), 251.
Allchin, Dr. W. H. [Ed.]—A manual of medicine (R.), 151.
American medicine (R.), 253.
American Physicians, Transactions of the Association of (R.), 165.
Anæmia, Pernicious, 236, 336.
Anæmia, Pernicious—Dr. W. Hunter (R.), 153.
Anæmia, Splenic, 235.
Anæmia, The metabolism in severe, 337.
Anæmias with leucopenia, 336.
Anæsthesia, The asphyxial factor in—H. B. Gardner (R.), 271.
Anæsthesia, The quiet production of—J. Freeman, 321.
Anæsthetics' committee of British Medical Association, Report of (R.), 270.
Aneurysm, A case of paralysis of right vocal cord due to thoracic—Dr. B. J. Baron, 208.
Angioma—The late Dr. J. Duncan (R.), 256.
Antiseptic club, Transactions of—A. Abrams (R.), 272.
Appendicitis, 51.
Appendicitis—C. B. Lockwood (R.), 248.
Appendicitis, Complications of, 237.
Arsenical poisioning in beer drinkers—Dr. T. N. Kelynack and W. Kirkby (R.), 254.
Artificial anus following herniotomy, A case of—A. W. Prichard, 119.
Ashby and G. A. Wright, Drs. H.—Diseases of children (R.), 264.
Asthma—Dr. E. Kingscote (R.), 348.
Auld, Dr. A. G.—Selected researches in pathology (R.), 260.
Axilla, A case of presentation at full term of the—Dr. F. P. Elliott, 130.

Baby, Our—Mrs. L. Hewer (R.), 356.
Bacteriology of every-day practice—Dr. J. O. Symes (R.), 161.
Barclay, W. M.—Report on surgery, 55.
Baron, Dr. B. J.—A case of paralysis of the right vocal cord due to thoracic aneurysm, 208; The progress in the treatment of throat, nose and ear disease during the past twenty years 289.
Barr, Dr. T.—Manual of diseases of the ear (R.), 265.
Barton, J. K.—Records from general practice (R.), 253.
Beck, Dr. C.—Fractures (R.), 155.
Beer drinkers, Arsenical poisoning in—Dr. T. N. Kelynack and W. Kirkby (R.), 254.
Berry, J.—Diseases of the thyroid gland (R.), 349.
Bladder, Ulceration of the—E. H. Fenwick (R.), 70.
Blood and blood-pressure—Dr. G. Oliver (R.), 158.

Blood at high altitudes, 337.
Blood in urine, New test for, 338.
Blood-regeneration in chlorosis, 336.
Blood-vessels, Circular suture of, 145.
Böhm and M. von Davidoff, Drs. A. A.—A text-book of histology, including microscopic technic (R.), 162.
Brain, Comparative physiology of the—Dr. J. Loeb (R.), 346.
Braine-Hartnell, C. — The uses of sanatoria for the treatment of tuberculosis, 2.
Bristol Medical Library, Some things of general interest in the—L. M. Griffiths (R.), 273.
Bristol Medico-Chirurgical Society, 85, 173, 375.
British Medical Association meeting at Cheltenham, 276.
Bucharest, Annales de l'institut de pathologie de (R.), 261.
Burdett's hospitals and charities (R.), 272.
Burghard, W. W. Cheyne and F. F.—A manual of surgical treatment (R.), 245.
Butlin and Dr. W. G. Spencer, H. T. — Diseases of the tongue (R.), 256.

Cantlie, Dr. J.—Plague (R.), 75.
Carpenter, Dr. G. — Syphilis of children (R.), 263.
Carwardine, T. — Operative and practical surgery (R.), 72 ; Report on surgery, 237 ; Cystoscopy and ureteral catheterization, 303.
Castration as a remedy for prostatic hypertrophy, 141.
Cattle, The tuberculin test in—G. S. Pollard, 210.
Celli, A.—Malaria according to the new researches (R.), 73.
Cerebellum, Case of abscess of the—Dr. J. M. Clarke and C. A. Morton, 112.
Cerebral science — Dr. W. Wood (R.), 249.
Cerebro-spinal meningitis, Six cases of—Dr. E. H. E. Stack, 44.
Chapman, Dr. C. W.—Heart disease in childhood (R.), 252.
Cheadle, Dr W. B.—On some cirrhoses of the liver (R.), 164.
Cheyne and F. F. Burghard, W. W. —A manual of surgical treatment (R), 245.
Childhood, Heart disease in—Dr. C. W. Chapman (R.), 252.
Children, Diseases of — Drs. H. Ashby and G. A. Wright (R.), 264.
Children, Syphilis of — Dr. G. Carpenter (R.), 263.
Chloroform — Dr. E. Lawrie (R.), 270.
Chlorosis, Blood-regeneration in, 336.
Cirrhoses of the liver, On some—Dr. W. B. Cheadle (R.), 164.
Clarke, Dr. J. M.—Report on medicine, 334.
Clarke and C. A. Morton, Dr. J. M. —Case of abscess in the left lobe of the cerebellum, 112.
Clinical Society of London, Transactions of (R.), 361.
Columbia University—Studies from the department of pathology of the College of Physicians and Surgeons (R.), 263.
Consumption and other preventable diseases, Statistics concerning—Dr. W. H. Symons, 7.
Convalescent Home, The Queen Victoria Jubilee, 167.
Coomber, Obituary notice of the late Mr. Thomas, 173.
Corpuscles of the blood, Formation of the, 334.
Cotton, Dr. W.—X-ray photographs as pictures, 131.
Cowen, R. J.—Electricity in gynæcology (R.), 261.
Craniotomy for Jacksonian epilepsy —W. J. Greer, 42.
Cross, F R.—Report on ophthalmology, 61.
Cystoscopy—T. Carwardine, 303.

Davidoff, Drs. A. A. Böhm and M. von—A text-book of histology, including microscopic technic (R.), 162.
Davies, Dr. D. S.—Plague and diphtheria, 367.
Davis, Dr. A. E.—Refraction of the Eye (R.), 71.
Dental surgery—H. Sewill (R.), 357.
Dermatological Association, Transactions of the American (R.), 269.
Dermatology. Report on—Dr. H. Waldo, 241.
Diagnosis, Essentials of—Drs. S. Solis-Cohen and A. A. Eshner (R.), 65.
Dictionary, The student's medical—Dr. G. M. Gould (R.), 75.
Diphtheria, Plague and—Dr. D. S. Davies, 367.

Displacement of abdominal viscera, Some cases of—Dr. T. Fisher, 217.
Duncan, The late Dr. J.—Angioma and other papers (R.), 256.

Eade, Sir P.—The Norfolk and Norwich Hospital (R.), 250.
Ear, Diseases of the—Dr. T. Barr (R.), 265.
Eccles, W. M.—Hernia (R.), 351.
Eczema, 241.
Edinburgh medical journal (R.), 361.
Edinburgh Obstetrical Society, Transactions of the (R.), 262.
Editorial notes, 76, 167, 274, 363.
Egg-shell membrane, Craniotomy, with the implantation of—W. J. Greer, 42.
Electricity, Medical—Dr. H. L. Jones (R.), 259.
Elliott, Dr. F. P.—A case of presentation of the axilla at full term, 130.
Ellis, H.—Studies in the psychology of sex (R.), 347.
Epileptics, Care and treatment of—Dr. W. P. Letchworth (R.), 67.
Eshner, Drs. S. Solis-Cohen and A. A.—Essentials of diagnosis (R.), 65.
Eye, Diseases of the—Dr. H. R. Swanzy (R.), 264.
Eye, Refraction of the—Dr. A. E. Davis (R.), 71.
Eye-strain in health and disease—Dr. A. L. Ranney (R.), 71.

Femoral hernia, Radical cure of, 240.
Fenwick, E. H.—Ulceration of the bladder (R.), 70.
"Festschrift" in honor of Dr. Abraham Jacobi (R.), 66.
Fibroids, Pregnancy complicated with, 148.
Fibro-myomata, Uterine, 146.
"First aid"—Drs. F. J. Warwick and A. C. Tunstall (R.), 259.
Firth, J. L.—On the advisability of ligaturing the jugular vein in sigmoid-sinus thrombosis, 36.
Fisher, Dr. T.—Two cases of sarcoma of intestine, 29; Report on medicine, 51; Some cases of displacement of abdominal viscera, 210.
Fothergill, Dr. W. E.—Manual of midwifery (R.), 357.
Fox, Dr. G. H.—Photographic atlas of the diseases of the skin (R.), 354.
Fractures—Dr. C. Beck (R.), 155.
Freeman, J.—The quiet production of anæsthesia, 321.
Freyer, Dr. P. J.—Stricture of the urethra and enlargement of the prostate (R.), 352.
Fyffe, Obituary notice of the late Dr. W. J., 97.

Gardner, H. B.—The asphyxial factor in anæsthesia (R.), 271.
Gastric ulcer, The origin of—Dr. W. Gordon, 100.
General practice, Records from—J. K. Barton (R.), 253.
Glasgow hospital reports (R.), 273.
Glasson, Dr. C. J.—Motherhood (R.), 356.
Gordon, Dr. W.—The origin of gastric ulcer, 100.
Gould, Dr. G. M.—The student's medical dictionary (R.), 75.
Goulstonian lectures on typhoid—Dr. P. Horton-Smith (R.), 256.
Grafstrom, Dr. A. V.—Medical gymnastics, including the Schott (Nauheim) movements (R.), 70.
Greer, W. J.—Craniotomy, with implantation of egg-shell membrane, 42.
Griffiths, L. M.—Some things of general interest in the Bristol Medical Library (R.), 273.
Guttmann, Dr. H.—Arzneiverordnungen in der kinderpraxis (R.), 263.
Guy's hospital reports (R.), 273.
Gymnastics, Medical—Dr. A. V. Grafstrom (R.), 70.
Gynæcological operations—Dr. S. Keith (R.), 262.
Gynæcology, Electricity in—R. J. Cowen (R.), 261.
Gynæcology, Points of practical interest in—Dr. H. Macnaughton-Jones (R.), 353.

Hæmoglobin, Formation of red, 334.
Hall, Drs. F. de H. and H. Tilley—Diseases of the nose and throat (R.), 266.
Hall, Dr. I. W.—Golden rules of physiology (R.), 163.
Harrison, R.—Vasectomy and urethro-stenosis (R.), 255.
Harsant, W. H.—Report on otology, 343.

Harveian lectures on pulmonary tuberculosis—Dr. R. Maguire (R.), 254.
Hawkins, C.—Physical examination of public school boys (R.), 264.
Heart-disease in childhood — Dr. C. W. Chapman (R.), 252.
Hemorrhages of pregnancy, On some of the—Dr. A. E. A. Lawrence, 196.
Herman, Dr. G. E.—Difficult labour (R.), 262.
Hernia—W. M. Eccles (R.), 351.
Hewer, Mrs. L.—Our Baby (R.), 356.
Hime, Dr. T. W.—Practical guide to the public health acts (R.), 268.
Histology, A text-book of—Drs. A. A. Böhm and M. von Davidoff (R.), 162.
Hoarseness and loss of voice, The clinical significance of—Dr. P. W. Williams, 203.
Hœpli, Manuali (R.), 257.
Horton-Smith, Dr. P.—Goulstonian lectures on typhoid (R.), 256.
Hospitals and charities, Burdett's (R.), 272.
Hunter, Dr. W.—Pernicious anæmia (R.), 153.
Huxley, T. H.—Lessons in elementary physiology (R.), 251.
Hygiene and public health—Drs. L. Parkes and H. Kenwood (R.), 356.
Hygiene, The journal of (R.), 271.

Index-catalogue of the library of the Surgeon-General's office, United States Army (R.), 362.
Infirmary, Bristol Royal, 364, 379.
Influenza, Leucopenia in, 335.
In Memoriam—Queen Victoria, 1.
Insurance, Life, 278.
International Congress of Medicine, 286, 382.
Ireland, Transactions of the Royal Academy of Medicine in (R.), 165.
Iron in blood formation, The rôle of, 335.

Jacksonian epilepsy, Craniotomy in —W. J. Greer, 36.
Jacobi, "Festschrift" in honor of Dr. Abraham (R.), 66.
Johns Hopkins hospital reports (R.), 74, 164.
Jones, Dr. H. L.—Medical electricity (R.), 259.
Jones, H. Macnaughton-; *see* Macnaughton-Jones.

Keetley, C. B.—Orthopædic surgery (R.), 68.
Keith, Dr. S.—Gynæcological operations (R.), 262.
Kelynack and W. Kirkby, Dr. T. N. — Arsenical poisoning in beer drinkers (R.), 254.
Kendall, Obituary notice of the late Dr., 381.
Kenwood, Drs. L. Parkes and H.—Hygiene and public health (R.), 356.
Kinderpraxis, Arzneiverordnungen in der—Dr. H. Guttmann (R.), 263.
Kingscote, Dr. E. — Asthma (R.), 348.
Kirkby, Dr. T. N. Kelynack and W. — Arsenical poisoning in beer drinkers (R.), 254.

Labour, Difficult—Dr. G. E. Herman (R.), 262.
Lake, R.—Laryngeal phthisis (R.), 267.
Laryngeal phthisis—R. Lake (R.), 267.
Laryngological Association, Transactions of the American (R.), 361.
Laryngology, Report on, 341.
Larynx, Tuberculosis of the, 342.
Lawrence, Obituary notice of the late Dr. A. E. Aust, 194.
Lawrence, Dr. A. E. Aust—On some of the hemorrhages of pregnancy, 196.
Lawrie, Dr. E.—Chloroform (R.), 270.
Lead, A case of muscular atrophy due to—Dr. J. E. Shaw, 117.
Leftwich, Dr. R. W.—An index of symptoms (R.), 254.
Letchworth, Dr. W. P.—Care and treatment of epileptics (R.), 67.
Leucocytosis, 234.
Libraries, Medical, 284.
Library of the Bristol Medico-Chirurgical Society, 91, 183, 282, 373.
Lichen ruber, 243.
Ligaturing the jugular vein in sigmoid-sinus thrombosis — J. L. Firth, 36.
Liver, On some cirrhoses of the—Dr. W. B. Cheadle (R.), 164
Local medical notes, 83, 187, 286, 379.
Lockwood, C. B.—Appendicitis (R.), 248.
Loeb, Dr. J.—Comparative physiology of the brain (R.), 346.

MacCormac, Obituary notice of the late Sir W., 366.

Macnaughton-Jones, Dr. H.—Points of practical interest in gynæcology (R.), 353.
Maguire, Dr. R.—Harveian lectures on pulmonary tuberculosis (R.), 254.
Malaria according to the new researches—A. Celli (R.), 73.
Manuali Hoepli (R.), 257.
Martindale and Dr. W. W. Westcott, W.—The extra pharmacopœia (R.), 257.
Mastoid operation, The complete, 343.
Materia medica and therapeutics—Dr. J. V. Shoemaker (R.), 358.
Meckel's diverticulum, Some surgical aspects of—Dr. W. Sheen, 310.
Medical annual (R.), 166.
Medical Society of London, Transactions of the (R.), 362.
Medicine, A manual of—Dr. W. H. Allchin [Ed.] (R.), 151.
Medicine, Cyclopædia of—Dr. C. E. Sajous (R.), 348.
Medicine, Reports on—Dr. J. M. Clarke, 334; Dr. T. Fisher, 51; Dr. G. Parker, 234; Dr. R. S. Smith, 136.
Medico-chirurgical transactions (R.), 362.
Midwifery, Manual of—Dr. W. E. Fothergill (R.), 357.
Morton, Dr. J. M Clarke and C. A.—Case of abscess in the left lateral lobe of the cerebellum, 112.
Motherhood—Dr. C. J. Glasson (R.), 356.
Murray, Dr. G. R.—Diseases of the thyroid gland (R.), 250.
Murrell, Dr. W.—What to do in cases of poisoning (R.), 258.
Muscular atrophy due to lead, A case of—Dr. J. E. Shaw, 117.

Nasal obstruction, Lectures on—Dr. A. M. Sheild (R.), 266.
Nasal respiration, 341.
Neuroma and neuro-fibromatosis—A. Thomson (R.), 66.
New-born, Red blood corpuscles and hæmoglobin in the, 335.
Newport New Hospital, 366, 380
New York, Transactions of the Medical Society of (R.), 361.
Norfolk and Norwich Hospital—Sir P. Eade (R.), 250.
Nose and throat, Diseases of the—Drs. F. de H. Hall and H. Tilley (R.), 266.
Nursing and hygiene, Illustrated lectures on—Dr. R. L. Roberts (R.), 74.

Obituary, 82, 97, 173, 193, 366, 381.
Obstetrics, Report on—Dr. W. C. Swayne, 146.
Ogden, Dr. J. B.—Clinical examination of the urine (R.), 349.
Oliver, Dr. G.—Blood and blood-pressure (R.), 158.
Operations on medical cases, Notes on—Dr. E. H. E Stack, 225, 326.
Operative and practical surgery—T. Carwardine (R.), 72.
Ophthalmological Society of the United Kingdom, Transactions of the (R.), 359.
Ophthalmological Society, Transactions of the American (R.), 265.
Ophthalmology, Report on—F. R. Cross, 61.
Organo-therapy, 170.
Orthopædic surgery—C. B. Keetley (R.), 68.
Otology, Report on, 343.

Pancreas, The surgery of the, 338.
Pancreatitis, 338.
Paralysis of right vocal cord due to thoracic aneurysm, A case of—Dr. B. J. Baron, 208.
Parker, Dr. G.—Report on medicine, 234.
Parkes, Drs. L. and H. Kenwood—Hygiene and public health (R.), 356.
Pathology, A text-book of—Dr. A. Stengel (R.), 260.
Pathology, Selected researches in—Dr. A. G. Auld (R.), 260.
Pediatric Society, Transactions of the American (R.), 264.
Pericardial effusion, Surgical treatment of, 144.
Pernicious anæmia, 236, 336.
Pernicious anæmia—Dr. W. Hunter (R.). 153.
Pharmacopœia, The extra—W. Martindale and Dr. W. W. Westcott (R.), 257.
Philadelphia, Transactions of the College of Physicians of (R.), 360.
Phillips, Dr. J.—Outline of the diseases of women (R.), 358.
Phthisis, Laryngeal—R. Lake (R.), 267.
Physiology, Golden rules of—Dr. I. W. Hall (R.), 163

Physiology, Lessons in elementary —T. H. Huxley (R.), 251.
Physiology, Manual of—Dr. G. N. Stewart (R.), 163.
Plague—Dr. J. Cantlie (R.), 75.
Plague and diphtheria—Dr. D. S. Davies, 367.
Poisoning, What to do in cases of—Dr. W. Murrell (R.), 258.
Pollard, G. S.—The tuberculin test in cattle, 210.
Pregnancy complicated with fibroids, 148.
Pregnancy, Leucocytes in, 335.
Pregnancy, On some of the hemorrhages of—Dr. A. E. A. Lawrence, 196.
Pregnant, Red blood corpuscles and hæmoglobin in the, 335.
Prichard, A. W.—Case of artificial anus following herniotomy, 119.
Prichard, Obituary notice of the late Dr. J. E., 82.
Prostate, Enlargement of the—Dr. P. J. Freyer (R.), 352.
Prostatic hypertrophy, Castration as a remedy for, 141
Public health acts, Practical guide to the—Dr. T. W. Hime (R.), 268.
Public Health Association, Reports of the American (R.), 269.
Public school boys, Physical examination of—C. Hawkins (R.), 264.
Pulmonary tuberculosis, Harveian lectures on—Dr. R. Maguire (R.), 254.
Purdy, Dr. C. W. — Practical uranalysis (R.), 252.

Queen Victoria—In Memoriam, 1.

Ranney, Dr. A. L.—Eye-strain in health and disease (R.), 71.
Red blood corpuscles and hæmoglobin in the pregnant and new-born, 335.
Refraction of the eye—Dr. A E. Davis (R.), 71.
Respiratory tract, Diseases of the upper—Dr. P. W. Williams (R.), 159.
Rheumatism, Acute, 136.
Rhinology, Report on, 341.
Roberts, Dr. R. L. — Illustrated lectures on nursing and hygiene (R.), 74.
Roentgen ray—Archives of the (R.), 260; X-ray photographs as pictures—Dr. W. Cotton, 131.
Saint Bartholomew's hospital reports (R.), 272.
Saint Thomas's hospital reports (R.), 273.
Sajous, Dr. C. E.—Cyclopædia of medicine (R.), 348.
Sanatoria for the treatment of tuberculosis, The uses of—C. Braine-Hartnell, 2.
Sanatorium for consumptives, Winsley, 168.
Sarcoma of the intestine, with secondary infection in one by a gas-forming bacillus, Two cases of—Dr. T. Fisher, 29.
Schott movements, Medical gymnastics, including the—Dr. A. V. Grafstrom (R.), 70.
Scraps, 95, 191, 286, 383.
Senn, Dr. N.—The pathology and surgical treatment of tumors (R.), 156.
Sewill, H. — Dental surgery (R.), 357.
Sex, Studies in the psychology of —H. Ellis (R.), 347.
Shaw, Dr. J. E.—A case of muscular atrophy due to lead, 117.
Sheen, Dr. W. — Some surgical aspects of Meckel's diverticulum, 310.
Sheild, Dr. A. M.—Lectures on nasal obstruction (R.), 266.
Shoemaker, Dr. J. V. — Materia medica and therapeutics (R.), 358.
Sick, Preparations for the, 79, 171, 280, 370.
Sigmoid-sinus thrombosis, Ligaturing the jugular vein in the treatment of—J. L. Firth, 36.
Skin, Photographic atlas of the diseases of the—Dr. G. H. Fox (R.), 354.
Skin practice, Golden rules of—Dr. D. Walsh (R.), 270.
Smith, Dr. R. S.—Report on medicine, 136.
Smithsonian report (R.), 360.
Soaps, 244.
Society, Bristol Medico-Chirurgical, 85, 173, 375.
Solis-Cohen and A. A. Eshner, Drs. S.—Essentials of diagnosis (R.), 65.
Spencer, H. T. Butlin and Dr. W. G.—Diseases of the tongue (R.), 256.
Splenic anæmia, 235.
Stack, Dr. E. H. E.—Six cases of cerebro-spinal meningitis, 44; Notes on operations on medical cases, 225, 326.

Stengel, Dr. A.—Text-book of pathology (R.), 260.
Stewart, Dr. G. N.—Manual of physiology (R.), 163.
Stock, Obituary notice of the late Mr. E., 382.
Surgery: its theory and practice—W. J. Walsham (R.), 255.
Surgery, Operative and practical—T. Carwardine (R.), 72.
Surgery, Reports on—W. M. Barclay, 55; T. Carwardine, 237; C. A. Morton, 141; Dr. J. Swain, 338.
Surgical treatment, A manual of—W. W. Cheyne and F. F. Burghard (R.), 245.
Sutherland, Dr. G. A.—Cyclic albuminuria (R.), 251.
Suture of the blood-vessels, Circular, 145.
Swain, Dr. J.—Retrospect of a third series of fifty consecutive intra-abdominal operations, 18, 121; Report on surgery, 338.
Swanzy, Dr. H. R.—Diseases of the eye (R.), 264.
Swayne, Dr. W. C.—Report on obstetrics, 146.
Symes, Dr. J. O.—The bacteriology of every-day practice (R.), 161.
Symons, Dr. W. H.—Statistics concerning consumption and other preventable diseases, 7.
Symptoms, An index of—Dr. R. W. Leftwich (R.), 254.
Syphilis of children—Dr. G. Carpenter (R.), 263.

Teeth, The cause and prevention of decay in—Dr. J. S. Wallace (R.), 355.
Tetanus treated by subcutaneous or intravenous injection, 143.
Thomson, A.—Neuroma (R.), 66.
Throat, Diseases of the nose and—Drs. F. de H. Hall and H. Tilley (R.), 266.
Throat, nose and ear during the past twenty years, The progress in the diagnosis and treatment of the—Dr. B. J. Baron, 289.
Thyroid gland, Diseases of the—J. Berry (R.), 349; Dr. G. R. Murray (R.), 250.
Tilley, Drs. F. de H. Hall and H.—Diseases of the nose and throat (R.), 266.
Tongue, Diseases of the—H. T. Butlin and Dr. W. G. Spencer (R.), 256.
Tubby, A. H.—Clinical lectures on intra-abdominal suppuration (R.), 350.
Tubercle, How to avoid—Dr. T. Wise (R.), 253.
Tuberculin test in cattle—G. S. Pollard, 210.
Tuberculosis, British Congress on, 170, 274, 366.
Tuberculosis of the larynx, 342.
Tuberculosis, The uses of sanatoria in treatment of—C. Braine-Hartnell, 2.
Tumors, The pathology and treatment of—Dr. N. Senn (R.), 156.
Tunstall, Drs. F. J. Warwick and A. C.—"First aid" (R.), 259.
Typhoid fever, Goulstonian lectures on—Dr. P. Horton-Smith (R.), 256.

University College, Bristol, 365; Faculty of Medicine, 363.
Upper respiratory tract, Diseases of the—Dr. P. W. Williams (R.), 159.
Uranalysis, Practical—Dr. C. W. Purdy (R.), 252.
Urethra, Stricture of the—Dr. P. J. Freyer (R.), 352.
Urine, Clinical examination of the—Dr. J. B. Ogden (R.), 349.
Urine, Test for blood in, 338.
Uterine fibro-myomata, 146.
Uterine tumours—W. R. Williams (R.), 352.

Vasectomy, 142.
Vasectomy and urethro-stenosis—R. Harrison (R.), 255
Visual tests in the services, 61.
Vocal cord, A case of paralysis of the right—Dr. B. J. Baron, 208.

Waldo, Dr. H.—Report on dermatology, 241.
Wallace, Dr. J. S.—Cause and prevention of decay in teeth (R.), 355.
Walsh, Dr. D.—Golden rules of skin practice (R.), 270.
Walsham, W. J.—Surgery: its theory and practice (R.), 255.
Warwick and A. C. Tunstall, Drs. F. J.—"First aid" (R.), 259.
Westcott, W. Martindale and Dr. W. W.—The extra pharmacopœia (R.), 257.

Wilding, Obituary notice of the late Dr. J., 173.
Williams, Dr. P. W.—The clinical significance of hoarseness and loss of voice, 203; Diseases of the upper respiratory tract (R.), 159; Report on laryngology and rhinology, 341.
Williams, W. R.—Uterine tumours (R.), 352.
Winsley sanatorium for consumptives, 168.
Wise, Dr. T.—How to avoid tubercle (R.), 253
Women, Diseases of—Dr. J. Phillips (R.), 358.
Wood, Dr. W.—Cerebral science (R.), 249.
Wright, Drs. H. Ashby and G. A.—Diseases of children (R.), 264.

X-rays—*see* Roentgen ray.

Year-book of the scientific and learned societies of Great Britain and Ireland (R.), 74.

---

J. W. Arrowsmith, Printer, Quay Street, Bristol.

